# Supplementary material for: Identification of photoperiod-induced specific miRNAs in the adrenal glands of Sunite sheep (Ovis aries)
Source: Front Vet Sci. 2022 Jul 22;9:888207. doi: 10.3389/fvets.2022.888207 (PMC9354845; doi:10.3389/fvets.2022.888207)
Supplement: Supplementary file 1 [file Data_Sheet_1.ZIP › Supplementary file/miRDeep2 file.zip!/miRDeep2 file/Know_Structure/LP42A2/oar-mir-370.pdf]

miRBase precursor : oar-mir-370
Total read count : 9493
oar-miR-370-5p read count : 28
oar-miR-370-3p read count : 9454
remaining reads : 11

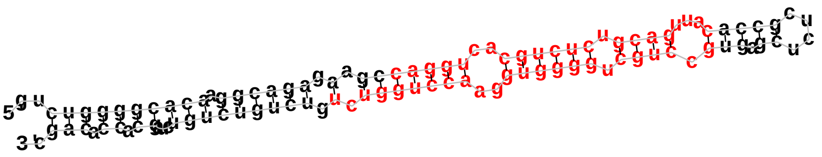

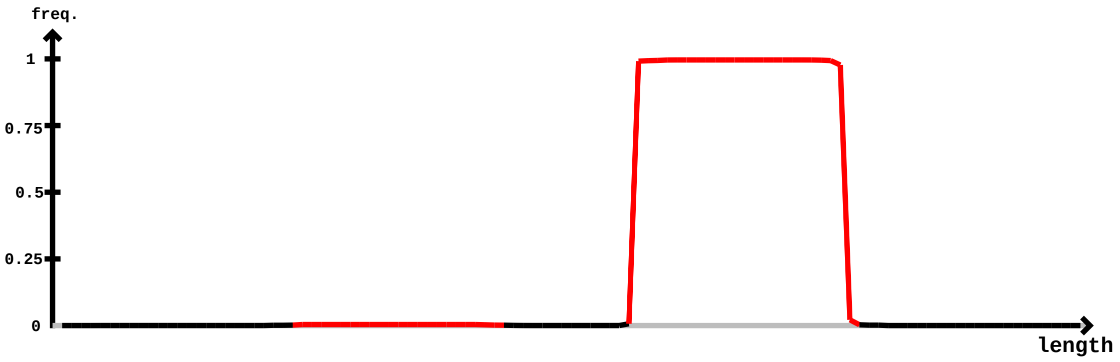

oar-miR-370-3p

oar-miR-370-5p

```
5'- gucuggggcacaaggcagagaagccaggucacgucucugcaguuacaccgcucucgagugccugcuggggguggaaccuggucugucugucugucuagcaccacagc   -3'    exp
    ..((((((((((.((((((.(.(((((((..((((((.((((...(((((....)).))).)))).))))))..))))))).).)))))))))...)).)).))).    reads      mm         sample
    ...uggggcacaaggcagagaagc...................................................................................    1          0          seq
    .....................agccaggucacgucucugcagu................................................................    8          0          seq
    .....................agccaggucacgucucugcaguu...............................................................    1          0          seq
    .......................ccaggucacgucucugcagu................................................................    1          0          seq
    .......................ccaggucacgucucugcaguu...............................................................    4          0          seq
    .......................ccaggucacgucucugcaguua..............................................................    1          0          seq
    ........................caggucacgucucugcaguu...............................................................    6          0          seq
    ........................caggucacgucucugcaguua..............................................................    3          0          seq
    ........................caggucacgucucugcaguuac.............................................................    7          0          seq
    ........................caggucacgucucugcaguuaca............................................................    4          0          seq
    ........................caggucacgucucugcaguuacac...........................................................    2          0          seq
    ................................................cgcucucgagugccugcuggggguggaaccuggu.........................    1          0          seq
    ..........................................................ugccugcuggggguggaaccu............................    2          0          seq
    ..........................................................ugccugcuggggguggaaccug...........................    1          0          seq
    ..........................................................ugccugcuggggguggaaccugg..........................    1          0          seq
    ..........................................................ugccugcuggggguggaaccuggu.........................    59         0          seq
    ..........................................................ugccugcuggggguggaaccugguc........................    1          0          seq
    ...........................................................gccugcuggggguggaacc.............................    1          0          seq
    ...........................................................gccugcuggggguggaaccu............................    5          0          seq
    ...........................................................gccugcuggggguggaaccug...........................    14         0          seq
    ...........................................................gccugcuggggguggaaccugg..........................    155        0          seq
    ...........................................................gccugcuggggguggaaccuggu.........................    9001       0          seq
    ...........................................................gccugcuggggguggaaccugguc........................    162        0          seq
    ...........................................................gccugcuggggguggaaccuggucu.......................    9          0          seq
    ............................................................ccugcuggggguggaaccuggu.........................    4          0          seq
    ............................................................ccugcuggggguggaaccugguc........................    10         0          seq
    ............................................................ccugcuggggguggaaccuggucu.......................    2          0          seq
    .............................................................cugcuggggguggaaccuggu.........................    4          0          seq
    .............................................................cugcuggggguggaaccugguc........................    3          0          seq
    .............................................................cugcuggggguggaaccuggucu.......................    2          0          seq
    ..............................................................ugcuggggguggaaccugguc........................    1          0          seq
    ..............................................................ugcuggggguggaaccuggucugu.....................    15         0          seq
    ...............................................................gcuggggguggaaccuggu.........................    2          0          seq
```
